# Roles of Arbuscular Mycorrhizal Fungi and Soil Abiotic Conditions in the Establishment of a Dry Grassland Community

**DOI:** 10.1371/journal.pone.0158925

**Published:** 2016-07-08

**Authors:** Jana Knappová, Hana Pánková, Zuzana Münzbergová

**Affiliations:** 1 Institute of Botany, Academy of Sciences, Zámek 1, 252 43 Průhonice, Czech Republic; 2 Department of Botany, Faculty of Science, Charles University in Prague, Benátská 2, 128 01 Prague, Czech Republic; Estación Experimental del Zaidín (CSIC), SPAIN

## Abstract

**Background:**

The importance of soil biota in the composition of mature plant communities is commonly acknowledged. In contrast, the role of soil biota in the early establishment of new plant communities and their relative importance for soil abiotic conditions are still poorly understood.

**Aims and Methods:**

The aim of this study was to understand the effects of soil origin and soil fungal communities on the composition of a newly established dry grassland plant community. We used soil from two different origins (dry grassland and abandoned field) with different pH and nutrient and mineral content. Grassland microcosms were established by sowing seeds of 54 species of dry grassland plants into the studied soils. To suppress soil fungi, half of the pots were regularly treated with fungicide. In this way, we studied the independent and combined effects of soil origin and soil community on the establishment of dry grassland communities.

**Key Results:**

The effect of suppressing the soil fungal community on the richness and composition of the plant communities was much stronger than the effect of soil origin. Contrary to our expectations, the effects of these two factors were largely additive, indicating the same degree of importance of soil fungal communities in the establishment of species-rich plant communities in the soils from both origins. The negative effect of suppressing soil fungi on species richness, however, occurred later in the soil from the abandoned field than in the soil from the grassland. This result likely occurred because the negative effects of the suppression of fungi in the field soil were caused mainly by changes in plant community composition and increased competition. In contrast, in the grassland soil, the absence of soil fungi was limiting for plants already at the early stages of their establishment, i.e., in the phases of germination and early recruitment. While fungicide affects not only arbuscular mycorrhizal fungi but also other biota, our data indicate that changes in the AMF communities are the most likely drivers of the observed changes. The effects of other soil biota, however, cannot be fully excluded.

**Conclusions:**

These results suggest that the availability of soil fungi may not be the most important limiting factor for the establishment of grassland species in abandoned fields if we manage to reduce the intensity of competition at these sites e.g., by mowing or grazing.

## Introduction

A proper understanding of the factors driving the establishment of plant communities on newly open substrates, such as various post-mining sites, dump sites or abandoned fields, is crucial for their effective management (e.g., [1–3]). Many studies have demonstrated that plant species diversity at such sites is strongly limited by the ability of species to disperse to such sites (e.g., [4–6]). However, even if plants arrive to the site (i.e., there is no dispersal or source limitation, see [5], [7], [8]), they may not be able to successfully recruit and survive at the sites because of unsuitable habitat conditions (i.e., habitat limitation, [9]). For example, former fields are often viewed as new potential habitats of species-rich grassland communities (e.g., [10–15]). However, many dry grassland species are absent from grasslands established on abandoned arable fields and are restricted to continuous grasslands (e.g., [16]) or colonize only a narrow edge of abandoned fields [17].

Habitat limitation, i.e., changes in soil biota and abiotic properties caused by former cultivation, is thought to be an important factor causing these impediments to recruitment. While many previous studies have demonstrated the key importance of soil abiotic properties (chemical and physical) for species establishment, individual plant performance and plant community structure, composition and diversity (e.g., [18–22]), the soil biota is also increasingly being recognized as an important driver of plant community composition (e.g., [23–30]).

Of the soil biota, arbuscular mycorrhizal fungi (AMF) have long been recognized as a key component affecting the growth of individual plants due to improved acquisition of nutrients (especially phosphorus) and water-induced stress tolerance in plants ([31–34]). AMF also have a strong impact on plant community structure [35] by changing plant competitiveness [36], thereby allowing the coexistence of many plant species (e.g., [37–40]), or by improving seedling establishment through their extensive mycelial network in the soil [41]. However, all of these effects of AMF are dependent on soil conditions and on the composition of the particular AMF community and their interactions (e.g., [39, 42]).

Soils of newly open areas, such as agricultural fields, usually contain lower numbers of AMF propagules ([43], [44]), have lower AMF diversity and are dominated by cosmopolitan species of AMF, which develop and sporulate very rapidly (e.g., [45], [46]). In contrast, slowly developing AMF species are less abundant or absent in such soils. Therefore, plant species may not be able to establish mycorrhizal associations with appropriate AMF species because these AMF species are absent from abandoned fields. Alternatively, grassland plants may be able to establish sufficient associations with field AMF, but the rapid colonization rate of these AMF supports competitively stronger plants (e.g., [47], [48]). Finally, the absence of plant species from abandoned fields may not be caused by AMF, but rather by altered abiotic soil properties. Distinguishing between these reasons for the absence of plants from abandoned fields could have strong impacts on the design of future projects aimed at restoring species-rich grasslands on abandoned fields.

Although some studies have focused on the joint effects of abiotic conditions and soil biota on the growth of individual plants (e.g., [42]) or on compositions of mature plant communities (e.g., [39]), such information for the *de novo* development of plant communities is still largely missing [49]. However, establishment of juvenile plants is a key phase determining the future development of plant communities, and small differences in seedling composition at early stages may have important effects on the composition and functions of plant communities at later stages of their development (e.g., [50–53]).

To study the effects of abiotic soil properties, soil biota and their interaction on the composition of a newly established grassland community, we set up microcosm in which we have sown 54 plant species. In half of the microcosms, we suppressed the development of soil fungi using the specific fungicide Karben Flo. This fungicide is based on the same active ingredient as Benomyl, which is the standard fungicide used in mycorrhizal studies because of its low phytotoxicity [54] but has not recently been produced [40]. We used soils from two different origins (dry grassland and abandoned field) with different pH and nutrient and mineral contents. We asked the following questions: 1) What is the relative importance of abiotic and biotic conditions and their interactions in the establishment of plant species and the final species diversity and composition of newly established dry grassland communities? and 2) What are the mechanisms affecting species responses to the suppression of the soil fungal community and the soil origin?

Twenty-eight grassland microcosms were established by sowing seeds from 54 species of dry grassland plants into both soils. To suppress soil fungal communities, half of the pots were regularly treated with fungicide. In this way, we studied the independent and combined effects of soil origin and fungal community on the establishment of dry grassland plant communities and the potential of soil from an abandoned field to host dry grassland plant communities. Our hypothesis was that the soil from the abandoned field would support species with lower mycorrhizal dependency, such as grasses, and that the effect of fungal suppression will thus be weaker in the soil from the abandoned field than in the dry grassland soil. In both types of soils, the suppression of fungal communities will lead to plant communities consisting of species with low mycorrhizal dependency. Obligatory mycorrhizal plants will grow better in the grassland soil than in soil from the abandoned field and will be absent in fungicide-treated pots.

## Methods

### Model species

The microcosm systems simulated calcareous dry grasslands in northern Bohemia, Czech Republic, Europe. Grasslands in this region form distinct localities surrounded primarily by agricultural fields; many of these agricultural fields have been abandoned and are currently undergoing succession towards dry grassland communities ([13], [55]). To set up the experimental dry grassland communities, we selected a heterogeneous group of 54 plant species representing a considerable portion of the grassland specialists typical of dry grasslands in Central Europe (S1 Table). For each pot (see below), we created a seed mixture consisting of 0.5 g of seeds from each species. In this way, we took into account the trade-off between seed size and seed germinability [56] and presumed that we gave each species an equal chance of germination. However, other experiments have used equal numbers of seeds rather than equal seed weight (e.g., [57], [58]) to set up their experimental communities. While we believe that our approach was better because we used many species with great inequalities in seed size (see also [53]), our methodology may not be the only correct approach. In any case, because we used the same sowing approach in all the pots and were primarily interested in the comparison among treatments, this decision does not have any major effects on the results of our study. In addition, our previous experiment [53] indicated that such a sowing strategy is suitable for creating communities with densities and compositions comparable to those in natural communities and that the species will thus experience natural levels of competition. The seeds were either collected in the field during summer and autumn 2008 or obtained from a local seed provider (Planta Naturalis, Czech Republic). The seeds collected in the field did not involve endangered or protected species and were collected at sites that are not private; thus, no specific permissions were required. Collection was performed in an area delimited by 50.5035683N, 14.2978586E; 50.5657611N, 14.2906486E; 50.4473233N, 13.9107619E and 50.5202872N, 13.8846694E. The seeds were stratified on moist filter paper in Petri dishes in the fridge at 4°C for 4 weeks in March 2009 before the experiment was established.

### Setup of the experiment

The experiment was established at the beginning of April 2009 in the experimental garden of the Institute of Botany, The Czech Academy of Sciences, Průhonice, Czech Republic (50°0′7.11″N, 14°33′20.66″E, 350 m asl, average annual temperature 8.8°C, mean annual precipitation 560 mm), and was regularly watered. We used 28 large circular pots (45 cm diameter, 30 cm height) to ensure a sufficiently large area and soil volume to mimic natural communities. In a previous study [53] we successfully used the same pots to establish and maintain species-rich dry grassland communities, indicating that our setup is suitable for the experiment. Half of the pots (14 pots) were filled with soil originating from a dry grassland, and the second half were filled with soil from a field abandoned approximately 10 years ago.

The two source locations (the dry grassland and the abandoned field) were 2 km apart in northern Bohemia, Czech Republic, approximately 70 km from the experimental garden. The soil was collected in February 2009 and transported to the experimental garden. During soil collection, the topsoil (approx. 5 cm), including plants, plant roots and possible seeds, was removed before soil extraction. Because percent root colonization (e.g. [59]), number of infective propagules (e.g. [60]), amount of spores (e.g., [45], [59]) and extraradical hyphae (e.g., [61]) were observed to decrease with soil depth, the upper soil layer (to approx. 15 cm) was stored in one set of bags and the lower soil layer (approx. between 15 and 30 cm) was stored in the other set of bags. The soil was placed into the experimental pots just after transport, with the lower layer of soil placed on the bottom of the pots and the upper layer of soil placed on the top. In this way, we tried to ensure that the soil coming into first contact with germinating seedlings will contain sufficient AMF.

The seeds were sown into the pots at the beginning of April 2009. All the pots were kept in the experimental garden and regularly watered. To avoid contamination of the pots with fungi from the surrounding environment, each pot in both treatment and control groups was surrounded by a cloth barrier attached to the edges of the pots that was 80 cm in height (S1 Fig).

### Fungicide application

To create a fully factorial experimental design, half of the pots (7 pots) within each of the two soil origins were treated with fungicide to suppress soil fungal communities. The fungicide was applied in February 2009 before experimental setup to suppress the germination of fungal spores prior to sowing the seeds. The fungicide was then applied monthly throughout the growing season (i.e., from March to November) until termination of the experiment in September 2010 according to the manufacturer’s instructions [62]. Monthly application of fungicide in an established grassland was previously shown to have strong effects on soil fungal communities and plant community composition (e.g., [40]). In agreement with Dostálek et al. [40], we used the fungicide Karben Flo Stefes [62] in the experiment. This fungicide contains the same active ingredient (carbendazim) as the formerly used fungicide benomyl, which has not been manufactured since 2001 [63]. Benomyl was previously the fungicide most widely utilized to manipulate AMF communities in field experiments, although it is not specific to AMF and can also influence certain non-target organisms, such as soil pathogenic fungi ([54], [64]) and other organisms, such as bacteria (e.g., [65–68]), or other components of the soil biota (e.g., [69]). Similar to changes in the AMF community, changes in the communities of these organisms could have significant effects on the performances of the plants in our microcosms. To separate the effects of pathogen suppression and the suppression of symbiotic AMF, we conducted an additional experiment with the same fungicide dosage in sterilized soil and the addition of AMF and soil pathogens, as well as their combination. This experiment showed that although Karben also suppressed pathogenic fungi in the soil, the positive effects from this suppression were minimal in comparison with the importance of AMF (Pánková et al. in prep.). Similarly, Newsham et al. ([54]) showed that for overall plant fitness, the positive effects from symbiotic endophytes are more important than those from the suppression of soil pathogens. We thus assume that most of the changes observed are due to changes in AMF communities. To support this expectation, we also evaluated the presence of pathogens and *Rhizobia* in roots of the plants as described below. However, when interpreting the results it is important to keep in mind that we are, in fact, observing changes in overall soil biota.

A total of 100 ml of the fungicide Karben Flo Stefes was diluted in 3 liters of distilled water and applied to treated pots. The control pots obtained the same amount of water as the treated pots. The dosage used in this experiment corresponded to that used in our previous field study [40].

### Data collection

#### Plant community

To record the species compositions in the pots, we harvested aboveground biomass (3 cm aboveground) in September 2009 and 2010. Such an approach allowed for the determination of biomass each year and simulated mowing, which is a common management practice in dry grasslands. The biomass was sorted into species, dried to a constant weight and weighed.

Part of the biomass of two species (*Brachypodium pinnatum* and *Salvia verticillata* in 2009 and *Centaurea jacea* and *Salvia verticillata* in 2010; these species were selected because they had sufficient biomass in all pots) from each pot was kept separate and used to analyze the nutrient contents of the biomass. The samples were dried in an oven at 80°C and the dried leaves were homogenized in a grinding mill and used to determine the phosphorus, nitrogen and carbon contents of the biomass. The nitrogen and carbon contents of the biomass were analyzed as described by Ehrenberger and Gorbach (1973). The phosphorus content was analyzed spectrophotometrically at a wavelength of 630 nm (Unicam UV4-100, Cambridge, UK; [70]) after digestion in HNO_3_ and H_2_O_2_.

#### Mycorrhizal parameters (biotic conditions)

The experiment was terminated after harvest in September 2010. All roots and associated soil were extracted from the pots, and the soil on the roots was washed away. The roots were sorted into species, and root samples of species with sufficient root biomass were used to evaluate root colonization (S1 Table). The finest roots were washed and stained with 0.05% trypan blue in lactoglycerol [71]. Colonization was assessed using the modified segment method [72] under a compound light microscope at 200 × magnification. The presence/absence of pathogens (all species) and *Rhizobia* (for Fabaceae only) in the roots were also evaluated.

To evaluate the effect of fungicide on AMF, we estimated the mycorrhizal inoculation potential of the soil (MIP; the potential of AMF propagules present in the soil to colonize host plant roots) in each pot in September 2009 and 2010. To estimate MIP, we used a standard bioassay approach with maize (a universal AMF host) as a host plant [73]. To do this, we took 10 soil cores (1 cm in diameter, 10 cm deep) from the pots and mixed the soil. The soil was then diluted with γ-sterilized soil taken from the same locations at a ratio of 1:100 (v:v). The prepared soil mixture was used to fill 125 ml pots. One pre-germinated maize seed (*Zea mays* L. cv. TATO) was planted in each pot. Five replicate pots were established for each sample. The plants were placed in a temperature-controlled greenhouse for 6 weeks. At harvest, the roots were washed and stained with 0.05% trypan blue in lactoglycerol [71]. MIP was estimated as the percentage of the root length of the host plant colonized by AMF. Colonization was assessed using a gridline-intersect method with 200 intersects per sample [72] under a dissecting microscope at 40 × magnification.

#### Abiotic conditions

To analyze the chemical compositions of the soils, we collected soil cores adjacent to those used for estimating MIP. We used the soil to analyze contents of total carbon, carbon in carbonates, organic carbon, total nitrogen, available phosphorus and the pH. These analyses were conducted using the methods described in Pánková et al. [74].

### Data analysis

#### Plant species richness and composition

The data on plant species richness were analyzed using ANOVA with soil origin, fungicide, year and all their interactions as predictors. The species composition data were analyzed using multivariate linear gradient analysis (redundancy analysis, RDA; Canoco for Windows 4.5 [75]). Biomass data from all individual species were used as dependent variables, and soil origin, fungicide, year and all their interactions were used as predictors. The significance of each predictor was tested using a Monte Carlo permutation test with 499 permutations using a split-plot design. The two measurements of the same pot represented two split-plots forming a whole plot. When testing the effects of soil origin, fungicide and their interactions, only whole plots were permuted. When testing the effects of time, only split-plots were permuted within whole plots. When testing the interactions between time and soil origin and/or fungicide, both whole plots and split plots were permuted. All the tests were carried out in four different ways. These included analysis without any standardization and transformation, analysis using logarithmically transformed biomass data, analysis standardized by species (giving each species the same weight in the analyses, removing differences between small and large species) and analysis standardized by samples (giving each sample the same weight in the analyses, removing differences between samples having different amount of total biomass, [76]). All of the results from these different analyses were largely similar. However, we present the results from the two analyses that were the most different but still quite similar, showing two important aspects of the data. Specifically, we present the analyses without any standardization and transformation, as they best represent the true data. In addition, we also used the analyses after standardization by samples. Such standardization removed the large differences in total plant biomass between the soils of the two different origins and provided deeper insights into the effects of fungicide.

In the case of a significant effect of fungicide on the soil chemical composition, we repeated all the above tests evaluating the effects of fungicide and its interactions with soil origin and year, with soil chemical composition as a covariate.

#### Chemical composition of biomass and soil

To analyze the chemical composition of plant biomass, we used data from one species (*S*. *verticillata)* over two years. These data were used to evaluate the effects of fungicide, soil origin, year and all their interactions on the contents of phosphorus and nitrogen and the C/N ratio using ANOVA. In addition, we tested the effects of fungicide, soil origin, species and their interactions on the nutrient content in *B*. *pinnatum* and *S*. *verticillata* biomass in 2009 and in *C*. *jacea* and *S*. *verticillata* biomass in 2010.

To analyze the chemical composition of the soil, and to test the effects of fungicide, soil origin and their interactions on total carbon, carbon in carbonates, organic carbon, nitrogen, C/N ratio, phosphorus and pH, we used ANOVA.

#### Mycorrhizal inoculation potential and root colonization

The effects of fungicide, soil origin, year and all of their interactions on mycorrhizal inoculation potential (MIP) were tested using logistic regression. For this analysis, the numbers of segments with and without root colonization in each sample were linked using a cbind function. This new composite variable was then used as the dependent variable in the test.

To analyze the effects of soil origin, fungicide and species on root colonization in the experiment, we selected species with at least 5 individuals that were analyzed for root colonization in each treatment. Such data were available only for 9 different species (S1 Table) because many species did not grow in the fungicide treatments. To compare only the effect of soil origin on percent root colonization for a larger spectrum of plants, we also selected species with at least 5 individuals from each soil origin in control pots, i.e., in pots without fungicide application (35 plant species, S1 Table). As with the MIP data, the cbind function and logistic regression were used to analyze the data. A separate analysis was run for each species.

## Results

### Vegetation species richness and composition

**Fig 1:**
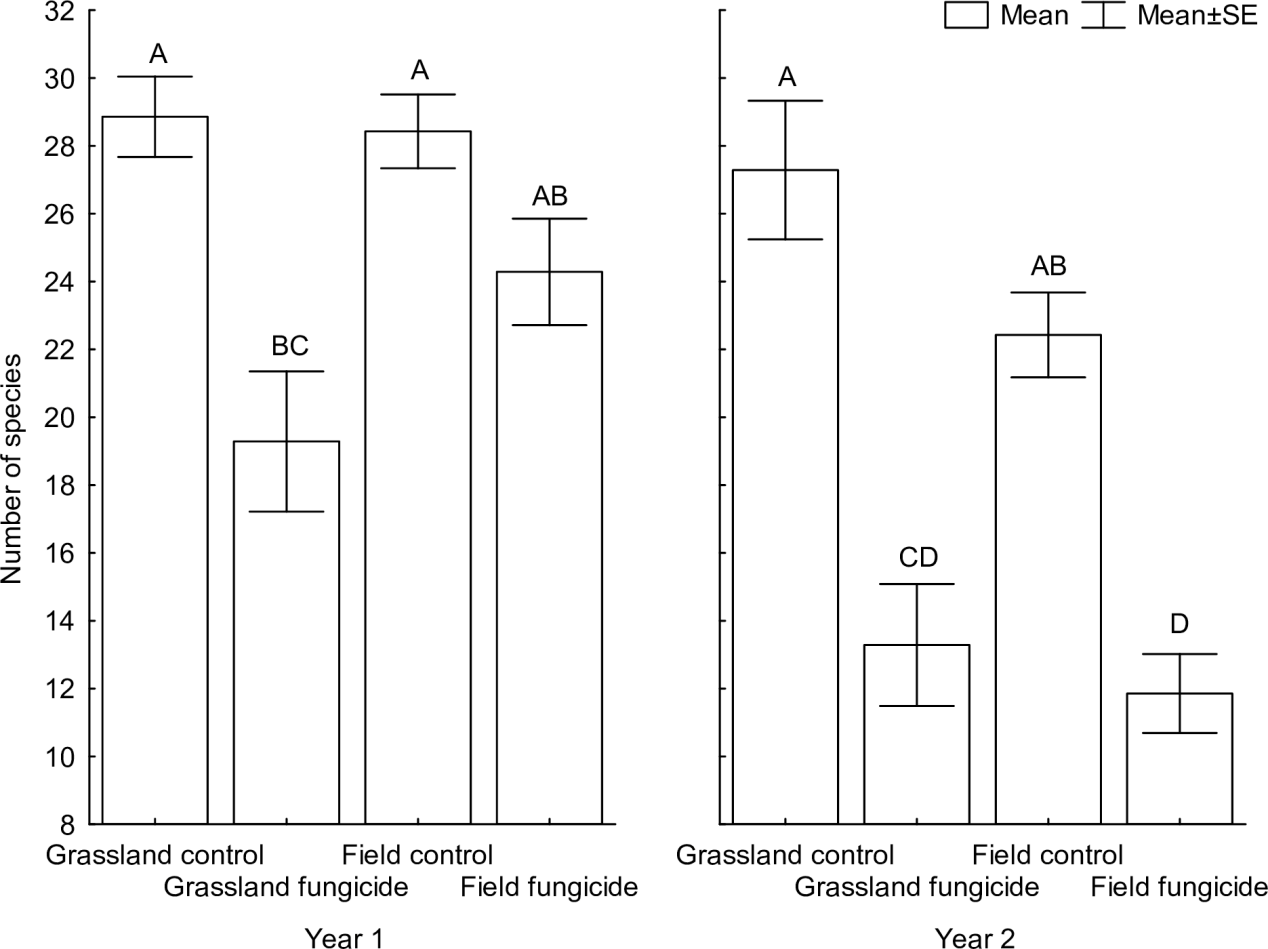
The effects of soil type, fungicide application and year on the number of species in the microcosms. Columns sharing the same letter are not significantly different (p > 0.05).

Fifty-one species germinated to become our experimental communities (S1 Table). Species richness was independent of soil origin (Table 1, detailed information in S2 Table, the data in S3 Table). However, species richness was significantly higher in the control treatment than in the fungicide treatment across all soils and years, except for field soil in year 1 (Fig 1). Species richness was also higher in the first year than in the second year (Table 1, detailed information in S2 Table). All of the two-way interactions among soil, fungicide and year, but not the three-way interaction, were significant (Table 1, detailed information in S2 Table). These interactions show that the decrease in species richness due to fungicide application was higher in the grassland soil. In the grassland soil, the number of species in the fungicide-treated pots decreased by 33% and 51% compared to untreated pots in the first and second years, respectively. In the field soil, the decrease was 15% in the first year and 47% in the second year (Fig 1). In addition, the number of species in the pots decreased over time and this decrease was significantly larger in the field soil and in the fungicide treatment (Fig 1, Table 1, detailed information in S2 Table). When soil abiotic properties were included as covariates in the analysis, the effects of fungicide and fungicide × year were still significant. All of the interactions with soil origin, however, became non-significant (Table 1, detailed information in S2 Table).

**Table 1:** The effects of soil type, fungicide and their interaction on plant species richness and plant species composition in the experiment. F-value for species richness and proportion of variance explained for species composition are shown. Significant values (p ≤ 0.05) are in bold and marked by *. Covar. indicates that abiotic characteristics of the soil were used as covariate. Df Error = 48. A full table including p-values is given in S2 Table.

	Standard.	Covar.	Soil	Fungicide	Year	Soil x fungicide	Soil x year	Fungicide x year	Soil x fungicide x year
Species richness		no	0.15	74.64*	34.42*	3.99*	6.00*	6.00*	0.20
		yes	0.02	66.16*	0.01	0.77	1.90	6.23*	0.25
Species comp.	No	no	0.34*	0.095*	0.11*	0.03*	0.05*	0.07	0.01
		yes		0.02*		0.003		0.01	0.01
	By sample	no	0.08*	0.23*	0.05*	0.03	0.02*	0.02*	0.02*
		yes		0.18*		0.01		0.02	0.01

**Fig 2:**
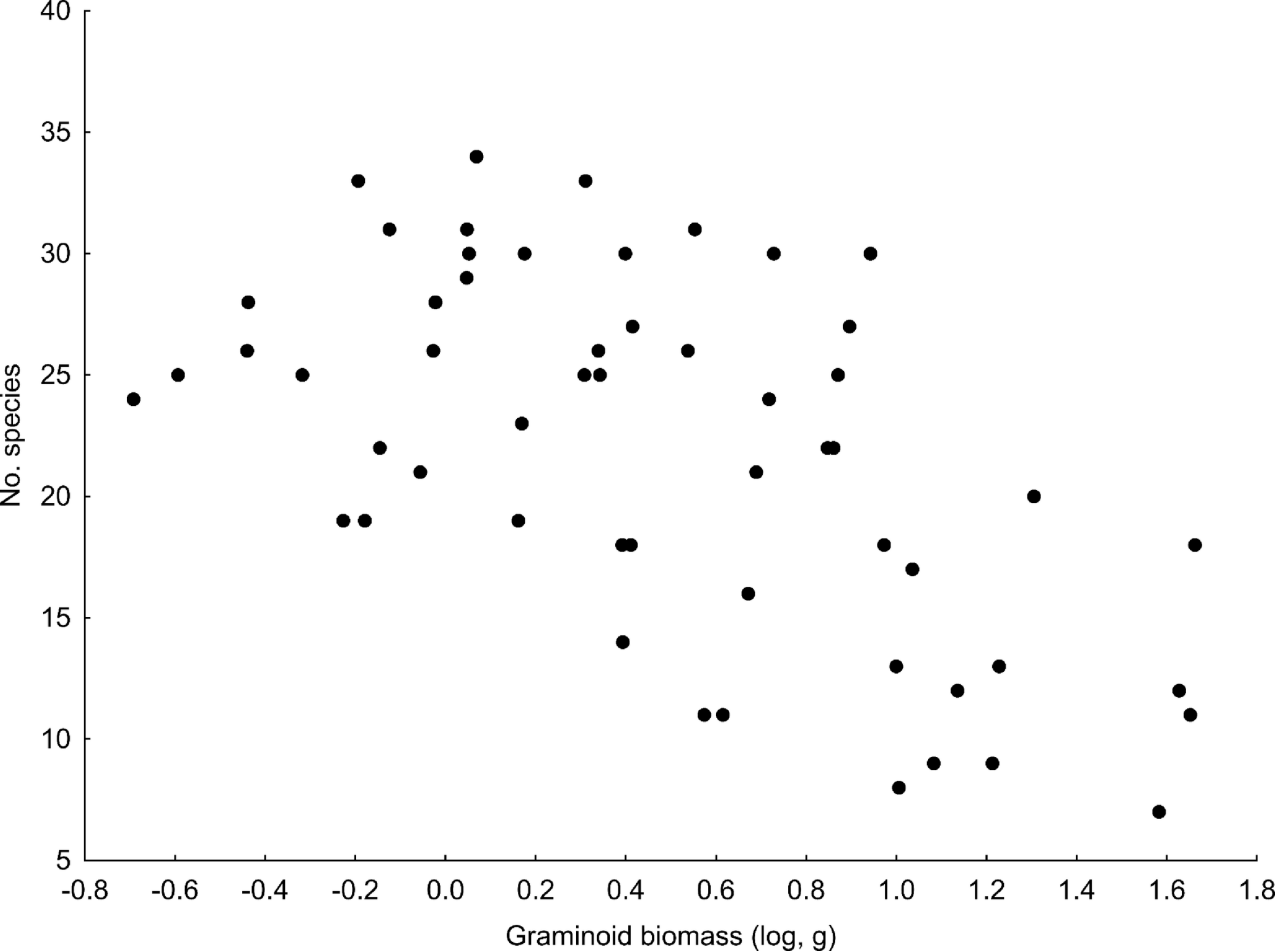
The relationship between the numbers of species in the microcosms and the biomass of the graminoids.

The number of species in the microcosms was strongly negatively correlated with the biomass of graminoids (df error = 55, r = -0.59, p < 0.001, Fig 2) and with total biomass (df error = 55, r = -0.27, p = 0.046).

**Fig 3:**
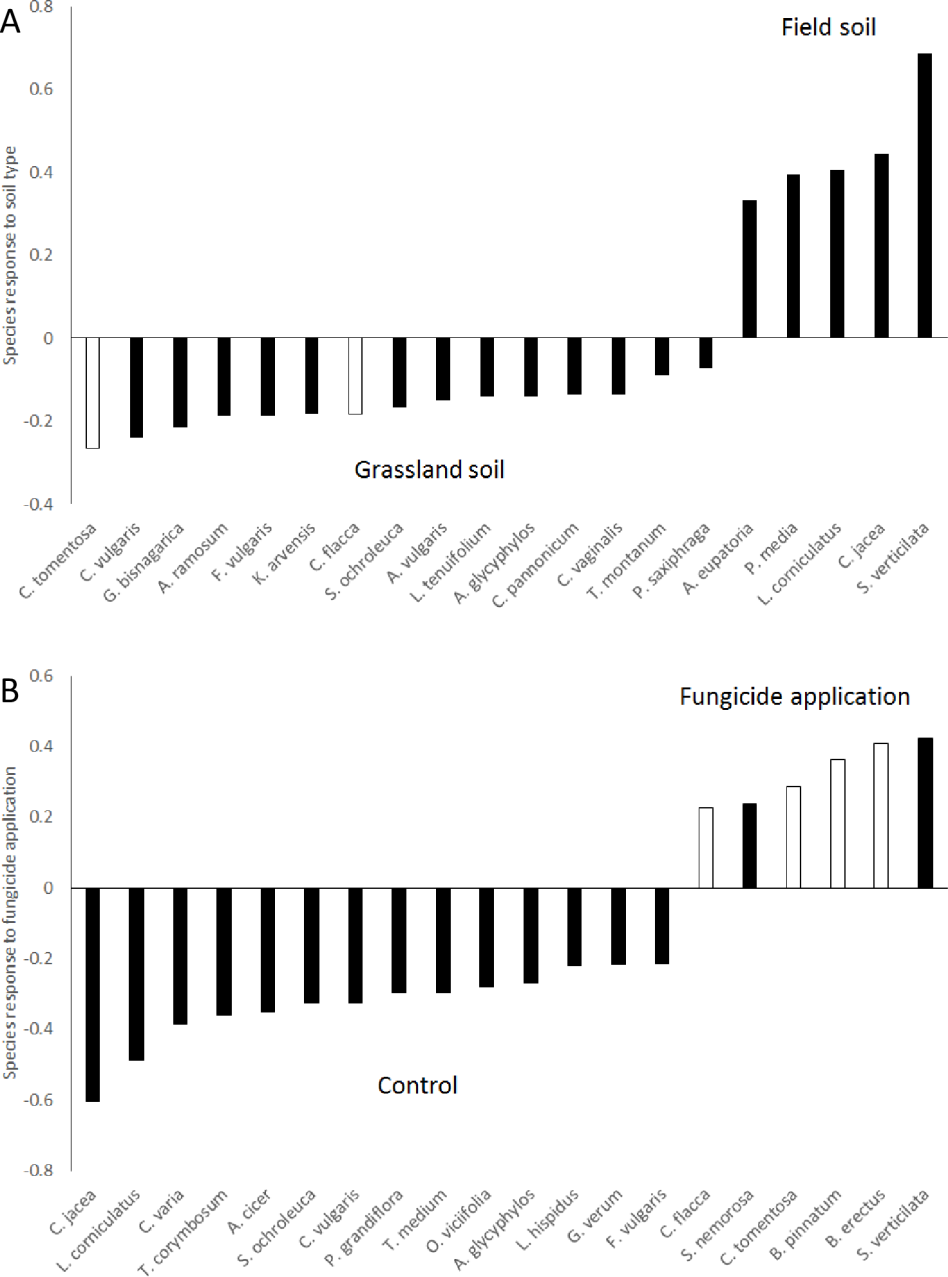
The effects of A) soil and B) fungicide on the species compositions of the communities. The response shows scores on the first constrained axis from RDA analysis A) without standardization and B) standardized by sample. Positive values show the affinity to A) field soil and B) fungicide application. Negative values show affinity to the A) grassland soil and B) controls. Twenty species with the strongest responses are shown. White columns show graminoids, i.e., plants from the families Poaceae and Cyperaceae.

Without standardization, almost 35% of the total variation in species composition was explained by soil origin. This clearly shows the strong effect of substrate on resulting plant communities. Interestingly, this effect did not occur only because all plants were larger in the field soil but also indicated that a wide range of plants grew better in the grassland soil (Fig 3A). However, the between-sample differences in total plant biomass were also important, as the percentage of variance explained decreased to 8% after standardization by samples (Table 1, detailed information in S2 Table). The species scores on the first ordination axis were largely correlated between these two analyses (R^2^ = 0.62), demonstrating that both analyses provided very similar insights into species preferences for soil origin. Similar to soil origin, the effect of year was stronger in the unstandardized data (Table 1, detailed information in S2 Table), and the species scores were largely correlated between the two analyses (R^2^ = 0.84).

In contrast to soil origin and year, the effect of fungicide was stronger after standardization by sample (Table 1, Fig 3B). Similar to the above analyses, the species scores were largely correlated between the two datasets (R^2^ = 0.86). Due to high correlations between species scores in the analyses with and without standardization, species responses to the treatments are shown only for the analyses using standardization by samples (Fig 3).

There was a weak significant interaction between soil origin and fungicide without standardization, and the interaction became non-significant after standardization. In contrast, a weak three-way interaction between soil origin, fungicide and year was significant only after standardization (Table 1, detailed information in S2 Table).

Because fungicide had a significant effect on the chemical composition of the soil (see below), we repeated the tests of the effects of fungicide and its interactions with other variables on plant species composition using soil abiotic properties as covariates. This analysis showed the effect of fungicide with and without standardization by sample was still significant, with only a minor reduction in the total variance explained (Table 1). In contrast, the previously significant interactions between fungicide and year and fungicide, soil origin and year became non-significant when soil characteristics were included as covariates (Table 1).

The species responding positively to the fungicide treatment were all of the graminoids included in the experiment (mainly *B*. *pinnatum*, *B*. *erectus*, *F*. *rubra*, *C*. *flacca* and *C*. *tomentosa*). The other positively responding species included two *Salvia* species (*S*. *verticillata* and *S*. *nemorosa*) and *Scorzonera hispanica*. Most other species grew better in the pots without fungicide application (Fig 3B). Species preferring the field soil included mainly tall herbs such as *A*. *eupatoria*, *C*. *glomerata*, *S*. *pratensis* and *S*. *verticillata*. Most other species were more common in the grassland soil (Fig 3A).

### Mycorrhizal inoculation potential of the soils and root colonization

**Fig 4:**
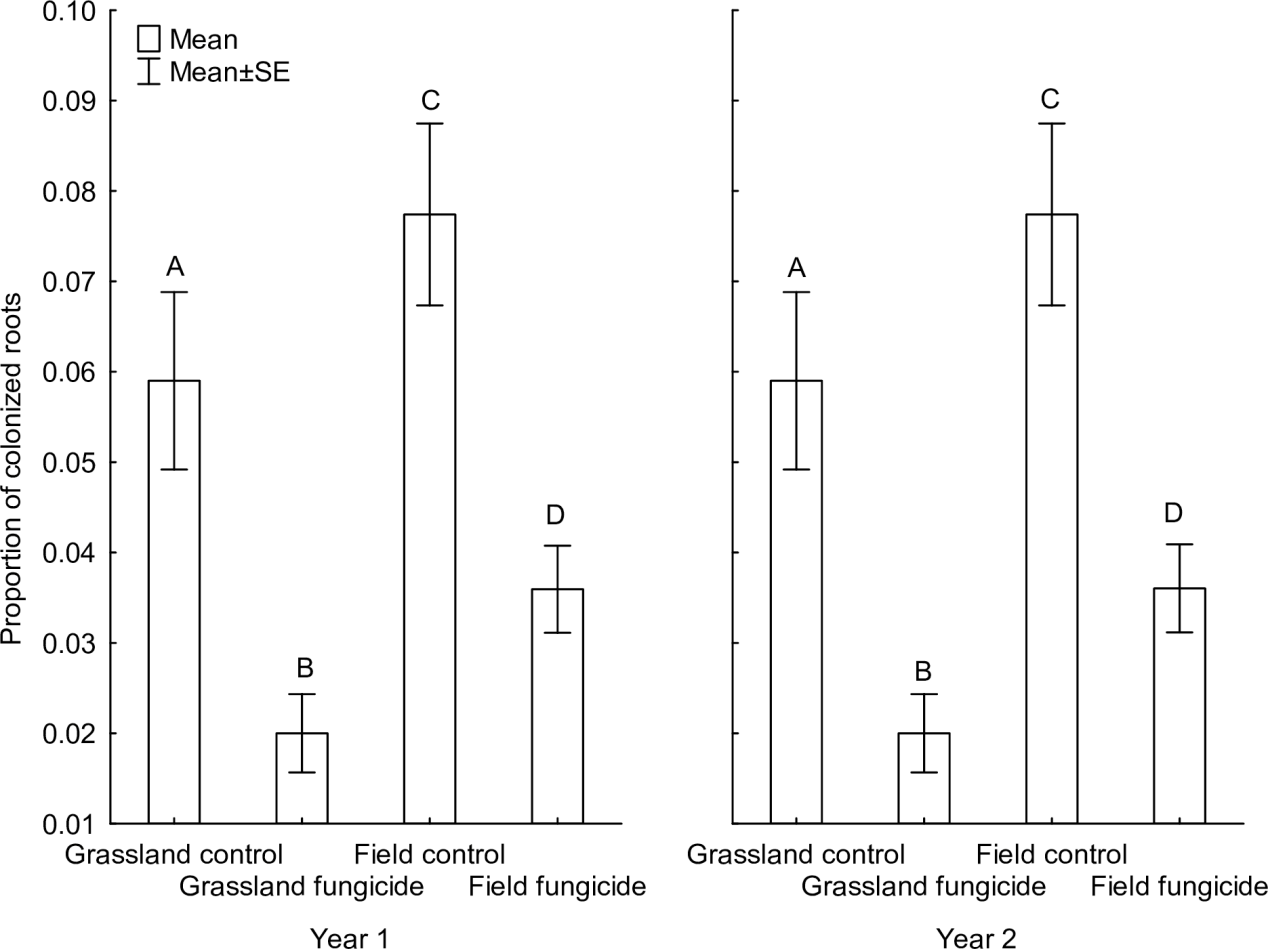
The effects of soil type, fungicide application and year on the proportion of the mycorrhizal inoculation potential of the soils described as the proportion of the roots colonized in *Zea* plants used as phytometer. Columns sharing the same letter are not significantly different (p > 0.05). The tests were performed using a generalized linear model assuming binomial distribution of the dependent variable. The dependent variable was represented by the number of colonized and uncolonized root segments linked by cbind function.

Soil mycorrhizal inoculation potential (MIP) was significantly affected by soil origin and fungicide, but not by year or any interaction among these variables (Table 2, detailed information is in S4 Table, the data in S5 Table). MIP was significantly higher in soils from the abandoned field and in soils without fungicide application than in soil from the dry grassland or soil with fungicide application (Fig 4).

**Table 2:** The effects of soil type, fungicide and year on mycorrhizal inoculation potential of the soil, on chemical composition of the soil and on nutrient content of biomass of *S*. *verticillata*. Df Error = 48 for all tests. The values are deviance for MIP and F-values for the other tests. Significant values (p ≤ 0.05) are in bold and marked by *. A full table including p-values is given in S3 Table.

		Soil	Fungicide	Year	Soil × fungicide	Soil × year	Fungicide × year	Soil × fungicide × year
Soil	MIP	133.68*	791.18*	<0.001	21.44	<0.001	<0.001	<0.001
	Total carbon	4523.76*	0.23	69.78*	5.69*	2.23	0.09	0.61
	pH	262.57*	45.63*	1.06	22.42*	2.27	1.28	1.87
	Nitrogen	1293.47*	25.58*	34.82*	15.47*	108.08*	0.71	0.71
	Carbon in carb.	24947.59*	0.06	111.35*	0.03	4.71*	0.12	0.08
	Organic carbon	20.87*	0.33	13.31*	5.83*	5.91*	0.20	0.44
	Phosphorus	19.57*	0.19	13.78	0.03	0.97	0.03	2.13
	C/N	781.78*	5.11*	77.28*	4.78*	92.36*	0.95	1.17
Biomass of *Salvia verticillata*	Phosphorus	0.55	22.01*	9.10*	2.42	0.58	1.25	0.41
	Nitrogen	0.37	63.06*	7.30*	3.48	0.07	5.73*	1.82
	C/N	0.01	50.44*	3.09	2.67	<0.001	0.85	0.68

In agreement with the results for MIP, nine plant species that were able to survive in both treatments had significantly lower root colonization in the fungicide treatment than plants in the control (df error = 427, dev. = 1801.41, p < 0.001). In contrast, soil origin did not have an overall effect on root colonization in plants across both treatments (df error = 427, dev. = 5.67, p = 0.52). However, the negative effect of fungicide on root colonization was stronger in the field soil than in the grassland soil (interaction soil type × fungicide, df error = 427, dev. = 59.87, p = 0.037). The effect of fungicide also significantly differed between species (interaction fungicide × species, df error = 427, dev. = 745.59, p < 0.001).

When tested separately for individual species, the effect of fungicide on root colonization was significant and negative across all species except for *B*. *pinnatum*. In contrast, the effect of soil origin was significant only for four species (Table 3, detailed information is in S6 Table, the data in S7 Table). For *B*. *pinnatum* and *S*. *hispanica*, root colonization was higher in the field soil. In contrast, *S*. *nemorosa* and *B*. *media* showed higher root colonization in the grassland soil. *B*. *media* was the only species showing a significant interaction between fungicide and soil origin, with the negative effect of fungicide being much stronger in the field soil (Table 3, detailed information is in S6 Table, the data in S7 Table).

**Table 3:** The effect of soil type, fungicide and their interaction on root colonization of plants in the experiment. The values shown represent deviance. Significant values (p ≤ 0.05) are in bold and marked by *. A full table including p-values and df-error is given in S5 Table and S6 Table.

	Soil	Fungicide	Soil x fungicide
*B*. *pinnatum*	39.40*	0.77	2.99
*B*. *erectus*	0.25	228.06*	17.20
*B*. *media*	33.94*	51.45*	47.17*
*C*. *jacea*	3.93	1076.67*	7.69
*C*. *scabiosa*	38.24	126.55*	40.85
*F*. *rupicola*	59.25	100.18*	22.19
*S*. *nemorosa*	93.16*	182.43*	0.12
*S*. *verticillata*	2.40	578.48*	0.77
*S*. *hispanica*	68.98*	158.14*	14.91

When comparing the percentage of root colonization in soils of different origins, the control pots without fungicide showed a significant effect of soil origin (df error = 563, dev. = 36.3, p < 0.001) and species (df error = 542, dev. = 6099.4, p < 0.001), as well as their interaction (df error = 521, dev. = 762,6, p < 0.001). The majority of species had higher root colonization in the soil from the abandoned field. Only four species (*C*. *varia*, *F*. *rupicola*, *P*. *media*, *P*. *grandiflora*) had higher root colonization in the soil from the grassland. No differences in the percentage of root colonization between the two soils were observed for six plant species (*B*. *media*, *C*. *glomerata*, *C*. *jacea*, *P*. *saxifraga*, *S*. *verticillata*, *T*. *pulegioides*).

There were no pathogens in the roots of most plant species. Pathogens were observed in up to three samples for *Salvia pratensis*, *S*. *verticillata*, *Sanguisorba minor*, *Pimpinela saxifraga* and *Campanula glomerata*. A higher occurrence of pathogens was observed only for *Lotus corniculatus* (11 samples out of 46). There was, however, no difference between pots treated with fungicide and control pots, nor between soils. Similarly, *Rhizobia* were abundant in the plant roots in both fungicide-treated and untreated pots, suggesting that fungicide did not have any effect on *Rhizobia* in roots.

### Chemical composition of plant biomass and soil

The tests analyzing nutrient content in plant biomass among all three species (*B*. *pinnatum*, *S*. *verticillata*, *C*. *jacea*) for each year separately and those using only *S*. *verticillata*, combining data from both years produced very similar results (results for *S*. *verticillata* are in Table 2, detailed information is available in S4 Table). Fungicide application decreased the content of phosphorus and the C/N ratio in the biomass and increased the content of nitrogen. There was no interaction effect between fungicide application and soil origin for any of the elements in the biomass (Table 2, detailed information is in S4, the data in S8 Table).

The test of the determinants of soil abiotic properties showed that the grassland soil had a significantly higher pH, contents of total carbon and carbon in carbonates, and C/N ratio. In contrast, the field soil had higher contents of nitrogen, organic carbon and phosphorus. Soil abiotic properties were also affected by fungicide application. Specifically, soil with fungicide application had a significantly lower pH, higher nitrogen content and a lower C/N ratio. The effects of fungicide significantly differed between the soil of the two origins. Specifically, fungicide had a stronger negative effect on pH in the field soil and a stronger negative effect on C/N in the grassland soil. In addition, it had a significantly stronger positive effect on nitrogen content in the field soil. For total and organic carbon, fungicide had a positive effect in the field soil and a weakly negative effect in the grassland soil (Table 2, detailed information is in S4, the data in S9 Table).

## Discussion

Our study clearly demonstrates that soil biota, most likely represented mainly by AMF, plays a crucial role in the diversity and composition of grassland communities and has even stronger effects than soil origin. Although fungicide application may affect pathogenic fungi in addition to AMF (e.g., [54], [64]) or bacteria, (e.g., [65–68]), our additional experiment (Pánková et al. in prep.) showed that the positive effects of the suppression of pathogenic fungi and bacteria were very low in comparison to the importance of AMF. Similarly, Newsham et al. [54] showed that for overall plant fitness, the benefits provided by symbiotic endophytes are more important than the benefits obtained from the suppression of soil pathogens. Because no differences in the presence of soil pathogens were found between treatments and because of the low impact of their suppression on plant growth, we interpret the data as effects of AMF. Nevertheless, in the case of legumes, the differences in plant growth could be caused not only by suppression of AMF but also by suppression of symbiotic bacteria from *Rhizobium* (e.g. [77], but see [78]). In our experiment, however, *Rhizobia* were found in roots of plants growing in pots treated and untreated with fungicide, and were thus unaffected by fungicide application. In addition, our data showed no differences in the abundance of soil pathogens in the roots of plants grown in the fungicide and control treatments, with an overall very low occurrence of pathogens in the roots.

We expected that the soil fungal community would play a much smaller role in the soils from the abandoned field than those from the grassland because the soil abiotic properties and soil biota were likely to be altered by previous agricultural practices [43]. Further, Voříšková et al. [79] showed that although abandoned fields host the same pool of AMF taxa as grasslands in the same region, the AMF community colonized plant roots faster from a lower number of infective propagules in the abandoned fields than in the grasslands. The results of our study, however, indicated that the suppression of soil fungal communities had an equal effect on the vegetation established in soils from both origins, and the effects of soil fungal communities and soil were largely additive. Contrary to our expectations, this result indicates that soil fungal communities, most likely represented mainly by AMF, are important to the same degree in the establishment of species-rich plant communities in both field and grassland soil.

### Species richness and vegetation composition

To properly demonstrate the effects of suppressing soil fungal communities on species richness and species composition, we considered the effects of the changed soil abiotic properties after fungicide application and used these properties as covariates. The amounts of variation in both species richness and composition explained by fungicide treatment surprisingly decreased very little when soil abiotic properties were included as covariates. This suggests that a reduction in soil fungal communities is a more important factor affecting plant species richness and community composition in the experimental microcosms than increased nutrient availability and decreased pH as a side effect of fungicide application (see also [40]).

The strong decrease in species richness due to suppression of soil fungal communities, especially in grassland soil in the first year and reaching over 50% in the second year, corresponds to our previous field study in the same dry grassland system [40]. Both results indicate that soil fungal communities, and primarily AMF, are important in the establishment of new communities as well as for stability of already existing plant communities. Such a strong effect in comparison to other studies (e.g., [65], [69], [80], [81]) can be related to the fact that the soil used in this experiment and the soil at the location used in Dostálek et al. [40] contain much lower amounts of phosphorus (grassland soil 4.4 mg/kg and field soil 6.1 mg/kg in our experiment) than the soils used in other studies and the standard agriculture soil from other areas (e.g., [18]). The beneficial effects of AMF on nutrient acquisition by the plants in the present study, as well as in our previous field study [40], are therefore likely to be much stronger than in comparable studies in soils containing more nutrients. The very strong effects of soil fungal community suppression on species richness in the grassland soil observed as soon as the first year suggest that AMF play a stronger role in the grassland soil than in the soil from the abandoned field, which is to be expected on the basis of nutrient content. On the other hand, a very strong decrease in species richness was also found in the second year in the soil from the abandoned field. This suggests that suppression of soil fungal communities can have large impacts on the establishment of the plant community in this soil as well, but the response of the plant community is slower. A possible explanation for this delayed effect may be the presence of more favorable conditions for initial seedling establishment and plant growth and stronger competition in the latter stages of community development. The differences in the timing of the effects of soil fungal community suppression on the plant communities could be explained by differences in life-history strategies in AMF inhabiting the different soils, as AMF colonized plant roots faster from a lower number of infective propagules in the abandoned field than in the grassland [79]. In contrast to dry grassland AMF, AMF from the abandoned field are rapid root colonizers because this soil has high MIP. Slow colonization rate in the grassland AMF could occur because at early stages of plant development, nutrient transport to the plants will be sufficient for slow-growing plants. However, these slow-colonizing AMF will not be able to support rapidly growing plants because their growth rate is faster than the ability of AMF to colonize their roots [82], and therefore these species will be limited in growth or may even be suppressed. On the other hand, AMF from the abandoned field are able to quickly colonize roots of all plants at the beginning of plant growth, but plants with fast growth rates will be favored because they are competitively stronger. Therefore, AMF suppression had stronger and immediate effects on the plant community in the grassland soil. In contrast, the effects of AMF suppression in the abandoned field were mediated by enhanced competition and were therefore delayed. The effect of soil origin on species composition was mainly linked to higher biomass in the soil from the abandoned field than to actual changes in species composition, as apparent from the comparison of analyses without standardization to those with standardization by samples. This contrasts with our previous microcosm study [53] and a range of other studies indicating that substrate is one of the key factors determining performance of individual plant species as well as composition of natural plant communities (e.g., [22], [83]). While it has been often suggested that small initial differences in composition of plant communities may have dramatic effects on later stages of community development (e.g., [51]), it is also possible that the differences between the two soils could increase over time due to stronger competitive interactions in the more nutrient-rich field soil [84].

While we expected that the effects of soil origin could change over time, the effects of suppressing soil fungal communities are largely congruent with our previous study [40] in the same system, which was instead performed in an established natural community. This indicates similar strong effects of AMF on the establishment of new plant communities and on the stability of already-existing plant communities. AMF thus support not only the performance of seedlings and adult plants [85], but also affect interspecific interactions such as competition (e.g., [86], [87]).

Species responses to the fungicide treatment also follow the general expectation that graminoids are less mycorrhizae-dependent and thus strongly benefit from fungicide application (e.g., [40], [88–91]). In agreement with Dostálek et al. [40] we also demonstrated a strong negative relationship between the biomass of graminoids and species richness. Importantly, this relationship was much stronger than the commonly explored relationship between total biomass and species richness (e.g., [92], [93]). This relationship could be explained by a higher root:shoot ratio and thus stronger belowground competition of graminoids compared to forbs [94]. Moreover, leaves of graminoid species also absorb specific portions of the photosynthetically active light spectrum and therefore specifically change conditions for growth of subordinate species [95].

Even though our results support the conclusion that species with lower dependency upon AMF are less affected by fungicide application, species response to AMF suppression interestingly was not correlated with root colonization of the plants in our control treatments (not shown). This could be explained by the fact that higher root colonization does not necessarily indicate higher functionality of the AMF symbiosis ([74], [96], [97]).

We also expected that species negatively responding to AMF suppression would be those that are naturally restricted to dry grasslands in the area, whereas species positively affected by fungicide treatment would occur in arable fields. This was based on the assumption that the fungal communities in arable fields tend to be suppressed due to a combination of ploughing, fertilization and pesticide and fungicide application ([98–101]). However, this assumption was not supported in this study, as species response to fungicide was not correlated with species affinity to arable fields, as reported in our previous study [55]. This lack of relationship may be explained by the fact that species distribution in arable fields is limited by other factors, such as the ability of a species to disperse to the fields and overcome intensive competition at the field locations (e.g., [14, 17]).

### Mycorrhizal inoculation potential of the soils and root colonization

All species experienced high root colonization in both soils not treated with fungicide. In line with our expectations based on the nutrient contents of the two soil types, root colonization was even higher in plants grown in grassland soil than in soils from the abandoned field. In the pots treated with fungicide, all plants had lower root colonization than in the control pots (except *Brachypodium pinnatum*). This result is in agreement with the results of MIP and confirms that the suppression of AMF was successful. The importance of AMF for plant growth, and especially for phosphorus acquisition by the plant, was also apparent from the decrease in phosphorus content in plant biomass after fungicide application. This further supported our expectation that fungicide application mainly caused a reduction in the AMF communities. The correlation between phosphorus concentration in plant biomass and the extent of AMF mycelia in the soil is commonly known (e.g., [32]). Similar effects of decreased phosphorus concentrations in biomass following a reduction in AMF were also demonstrated in other studies (e.g., [40], [102]).

Inoculation potential of the AMF community differed between the grassland soil and the soil from the abandoned field, with higher MIP values in the soil from the abandoned field. In line with this result, root colonization in the treatments without fungicide application was higher in the soil from the abandoned field than in the grassland soil for most species. This finding was surprising, because plants grown in the field at the same study sites experienced a consistently higher percent root colonization in the grassland than in the abandoned field [79]. Also, soil from the abandoned field contains more nutrients than the grassland soil and AMF symbiosis may therefore be less important. A possible explanation could be related to the combination of high MIP in the soil from the abandoned field (which was also observed under natural conditions [79]), elimination of stress factors such as drought in the garden, and the short duration of the experiment (only two years). The combination of these factors could allow plants with lower mycorrhizal colonization to avoid competitive exclusion from the plant community in the dry grassland soil over the two years, but these species could disappear at a later date. However, such plants would be quickly eliminated in nature. A further explanation could be that root colonization by the grassland AMF, which are thought to be slow colonizers ([46], [82]), was not fully complete at the time of harvest of the experiment.

The higher MIP of the soil from the abandoned field than the grassland soil is also in agreement with studies of Richter et al. [103] and Johnson et al. [47], showing higher MIP in disturbed soils. Similarly, such changes in AMF infectivity were observed during the succession of abandoned fields (e.g., [104], [105]). Higher infectivity of AMF from the abandoned field than from the grassland suggests that AMF from the abandoned field are better root colonizers with faster growth, as was also found in disturbed soils [48]. Such differences in the AMF community could be caused by the presence of different AMF species ([27], [100], [106], but see [46]) or by different responses of the same AMF community to particular environmental conditions (e.g., [79]).

Only nine species (mainly graminoids) were able to survive under the condition of reduced AMF. These species are considered to be facultatively dependent on AMF and therefore should be able to establish mycorrhizal symbioses with many AMF species. Although these species were colonized by AMF, the percentage was lower in the fungicide-treated pots than in the control pots or under natural conditions in the field. Fungicide application had a strong impact on root colonization by AMF as well as on the mycorrhizal inoculation potential of the soil (MIP), and is in line with data from previous studies (e.g., [40]). The effect of fungicide on root colonization was stronger in the field soil. This could be explained by the functionality of the applied fungicide, carbendazim, which inhibits development of germ tubes, formation of appressoria and growth of mycelia (AgrEvo), i.e., affects infection and growth traits of AMF. Different AMF species differ in these traits (e.g., [82], [107], [108]) and thus in their sensitivity to fungicide. In combination with the fact that the soils of the two different origins differ in their composition of AMF communities [79] and mycorrhizal inoculation potential, stronger effects of fungicide in the abandoned field could be explained by a higher sensitivity of local AMF species to the fungicide along with higher infectivity of its AMF community.

### Nutrients in plant biomass and in the soil

While it has been often demonstrated that fungicide application has no or weak effects on soil abiotic properties (e.g., [67], [109]), we found significant effects in the current study. The observed decrease in soil pH is likely a direct effect of the composition of the fungicide [40]. Similarly, the changes in nitrogen content could be caused by fungicide composition or could be related to the fact that the fungicide killed large amounts of fungal organisms and the dead biomass released high quantities of nitrogen into the soil, as suggested by Chen et al. [110]. The same explanation is likely the case for the increase in organic and total carbon content following fungicide application. The effect of fungicide on soil abiotic properties also strongly interacted with soil origin. All such effects, whether positive or negative, were stronger in the field soil, which is richer in nutrients and more acidic.

## Conclusions

To conclude, this study showed that soil fungi, likely represented mainly by AMF, are important in the establishment of dry grassland plants in both soil types. Soil fungal communities play even stronger roles than soil conditions and the effects of these two factors are additive. Contrary to our expectations, this result indicates that soil fungi are important to the same degree in the establishment of species-rich plant communities in both soil from the abandoned field and the grassland. The effects of the suppression of soil fungal communities on species richness, however, occurred less quickly in the soil from the abandoned field than in the grassland soil. This could be explained as an additive effect of different soil properties and life- history strategies of AMF communities and plants in the different soils. The negative effects of suppression of soil fungi in the field soil are caused mainly by changes in plant community composition and increased competition. In contrast, in the grassland soil, the absence of AMF is limiting for plants already in the early stages of their establishment. This could suggest that the availability of AMF may not be the key limiting factor for the establishment of grassland species in the abandoned field if the intensity of competition at these sites is reduced through management e.g., by mowing or grazing.

## Supporting Information

S1 Fig Experimental set up. A) Larger view over a set of the pots. Only 28 of these pots were used for the experiment. The remaining ones represented treatment that eventually failed and is not presented. B) Detailed view of one experimental pot.
(DOCX)

S1 Table List of species used in the experiment. Germinated indicates that the given species successfully germinated and was thus included in the experiment. Root colonization 1 indicates that we were able to use data on root colonization from fungicide untreated pots, 2 indicates that we had sufficient data from both pots with and without fungicide. The species names are unified according the online version of Flora Europaea http://rbg-web2.rbge.org.uk/FE/fe.html, accesses on March 3^rd^, 2014.
(DOCX)

S2 Table The effect of soil type, fungicide and their interaction on plant species richness and plant species composition in the experiment. Significant values (p ≤ 0.05) are in bold. Df Error = 48. For significant effect of soil type for species richness, F and G indicates higher value in the soil from abandoned field and grassland, respectively. For significant effect of fungicide, C and F indicates higher value in the soil from control and fungicide treated plots, respectively.
(DOCX)

S3 Table Primary data showing number of species in pots of the different treatments in the two years. (DOCX)

S4 Table The effect of soil type, fungicide and year on mycorrhizal inoculation potential of the soil, on chemical composition of the soil and on nutrient content of biomass of *S*. *verticilata*. Df Error = 48 for all tests. Significant values (p ≤ 0.05) are in bold. Dev. stands for Deviance. For significant effect of soil type, F and G indicates higher value in the soil from abandoned field and grassland, respectively. For significant effect of fungicide, C and F indicates higher value in the soil from control and fungicide treated plots, respectively.
(DOCX)

S5 Table Primary data showing root colonization in the mycorrhizal inoculation assessment in the pots of the different treatments. (DOCX)

S6 Table The effect of soil type, fungicide and their interaction on root colonization of plants in the experiment. Significant values (p ≤ 0.05) are in bold. For significant effect of soil type, F and G indicates higher value in the soil from abandoned field and grassland, respectively. For significant effect of fungicide, C and F indicates higher value in the soil from control and fungicide treated plots, respectively.
(DOCX)

S7 Table Proportional root colonization of plants in the experiment. The values are mean±SE of proportion of roots colonized.
(DOCX)

S8 Table Content of phosphorus, nitrogen and carbon to nitrogen ratio in biomass of selected species grown in the soil from the abandoned field and grassland, with and without fungicide application. The values are mean±SE.
(DOCX)

S9 Table Content of nitrogen, total and organic carbon and carbon in carbonates, phosphorus, pH and C/N ratio in soil from the abandoned field and grassland with and without fungicide application. The values are mean±SE.
(DOCX)
