# Supplementary material for: Roles of Arbuscular Mycorrhizal Fungi and Soil Abiotic Conditions in the Establishment of a Dry Grassland Community
Source: PLoS One. 2016 Jul 8;11(7):e0158925. doi: 10.1371/journal.pone.0158925 (PMC4938501; doi:10.1371/journal.pone.0158925)

S2 Figure. Experimental set up. A) Larger view over a set of the pots. Only 28 of these pots were used for the experiment. The remaining ones represented treatment that eventually failed and is not presented. B) Detailed view of one experimental pot.


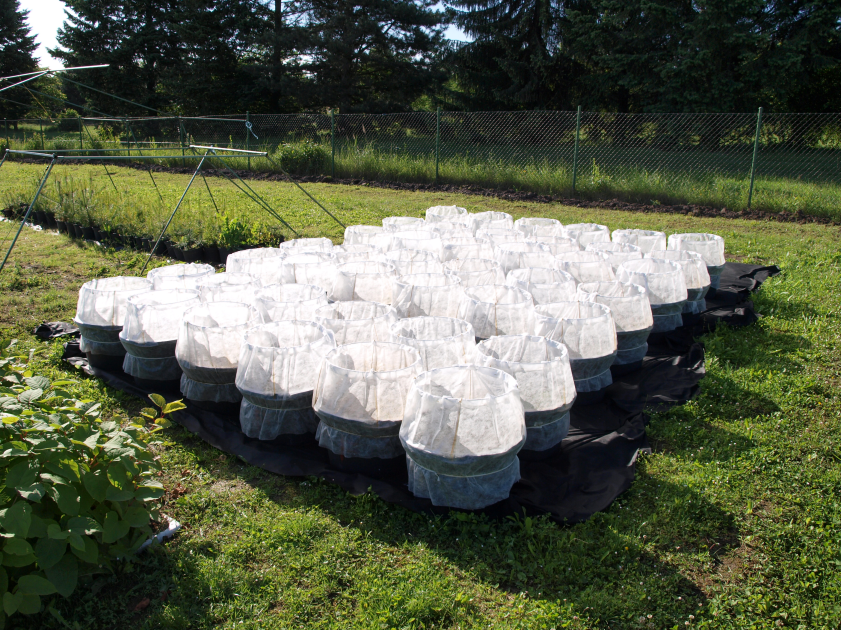


B)


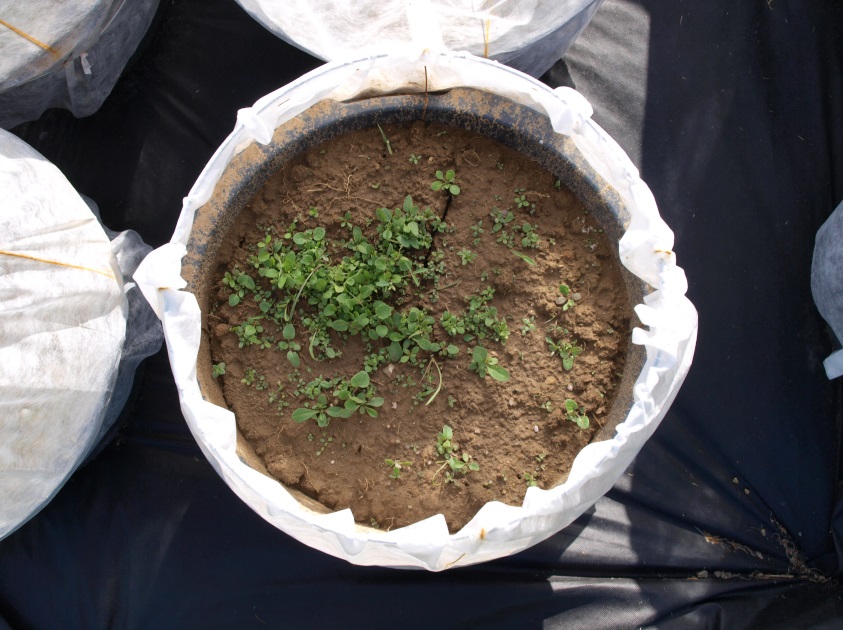

Supplement: S1 Fig — A) Larger view over a set of the pots. Only 28 of these pots were used for the experiment. The remaining ones represented treatment that eventually failed and is not presented. B) Detailed view of one experimental pot. (DOCX) [file pone.0158925.s001.docx]
