# Supplementary material for: Roles of Arbuscular Mycorrhizal Fungi and Soil Abiotic Conditions in the Establishment of a Dry Grassland Community
Source: PLoS One. 2016 Jul 8;11(7):e0158925. doi: 10.1371/journal.pone.0158925 (PMC4938501; doi:10.1371/journal.pone.0158925)
Supplement: S1 Table — Germinated indicates that the given species successfully germinated and was thus included in the experiment. Root colonization 1 indicates that we were able to use data on root colonization from fungicide untreated pots, 2 indicates that we had sufficient data from both pots with and without fungicide. The species names are unified according the online version of Flora Europaea http://rbg-web2.rbge.org.uk/FE/fe.html, accesses on March 3rd, 2014. (DOCX) [file pone.0158925.s002.docx]

S1 Table. List of species used in the experiment. Germinated indicates that the given species successfully germinated and was thus included in the experiment. Root colonization 1 indicates that we were able to use data on root colonization from fungicide untreated pots, 2 indicates that we had sufficient data from both pots with and without fungicide. The species names are unified according the online version of Flora Europaea <http://rbg-web2.rbge.org.uk/FE/fe.html>, accesses on March 3^rd^, 2014.

| Species | Family | Germinated | Root colonization |
| --- | --- | --- | --- |
| Agrimonia eupatorium | Rosaceae | 1 | 1 |
| Anthericum ramosum | Liliaceae | 1 |  |
| Anthylis vulneraria | Fabaceae | 1 |  |
| Asperula tinctoria | Rubiaceae | 1 |  |
| Aster amellus | Compositae |  |  |
| Astragalus cicer | Fabaceae | 1 | 1 |
| Astragalus glycyphylos | Fabaceae | 1 | 1 |
| Brachypodium pinnatum | Gramineae | 1 | 2 |
| Briza media | Gramineae | 1 | 2 |
| Bromus erectus | Gramineae | 1 | 2 |
| Bupleurum falcatum | Apiaceae | 1 | 1 |
| Campanula glomerata | Campanulaceae | 1 | 1 |
| Carex flaca | Cyperaceae | 1 | 1 |
| Carex tomentosa | Cyperaceae | 1 |  |
| Carlina vulgaris | Compositae | 1 | 1 |
| Centaurea jacea | Compositae | 1 | 2 |
| Centaurea scabiosa | Compositae | 1 | 2 |
| Cirsium acaule | Compositae | 1 |  |
| Cirsium pannonicum | Compositae | 1 |  |
| Coronilla vaginalis | Fabaceae | 1 |  |
| Coronilla varia | Fabaceae | 1 | 1 |
| Festuca rupicola | Gramineae | 1 | 2 |
| Filipendula vulgaris | Rosaceae | 1 |  |
| Galium verum | Rubiaceae | 1 | 1 |
| Gentiana cruciata | Gentianacea |  |  |
| Globularia punctata | Globulariaceae | 1 |  |
| Helianthemum grandiflorum | Cistaceae | 1 |  |
| Inula salicina | Compositae | 1 | 1 |
| Knautia arvensis | Dipsacaceae | 1 | 1 |
| Leontodon hispidus | Compositae | 1 | 1 |
| Linum flavum | Linaceae | 1 |  |
| Linum tenuifolium | Linaceae | 1 |  |
| Lotus corniculatus | Fabaceae | 1 | 1 |
| Onobrychis_viciifolia | Fabaceae | 1 | 1 |
| Ononis spinosa | Fabaceae | 1 | 1 |
| Peucedanum cervaria | Umbelliferae | 1 |  |
| Pimpinella saxifraga | Umbelliferae | 1 | 1 |
| Plantago media | Plantaginaceae | 1 | 1 |
| Potentilla heptaphyla | Rosaceae | 1 |  |
| Primula veris | Primulaceae |  |  |
| Prunela grandiflora | Lamiaceae | 1 | 1 |
| Salvia nemorosa | Lamiaceae | 1 | 2 |
| Salvia pratensis | Lamiaceae | 1 | 1 |
| Salvia verticilata | Lamiaceae | 1 | 2 |
| Sanquisorba minor | Rosaceae | 1 | 1 |
| Scabiosa ochroleuca | Dipsaceceae | 1 | 1 |
| Scorzonera hispanica | Compositae | 1 | 2 |
| Sesleria varia | Gramineae | 1 |  |
| Stachys recta | Lamiaceae | 1 | 1 |
| Tanacetum corymbosum | Compositae | 1 | 1 |
| Teucrium chamaedris | Lamiaceae | 1 | 1 |
| Thymus pulegioides | Lamiaceae | 1 | 1 |
| Trifolium montanum | Fabaceae | 1 |  |
| Trifolium medium | Fabaceae | 1 | 1 |
