# Supplementary material for: Roles of Arbuscular Mycorrhizal Fungi and Soil Abiotic Conditions in the Establishment of a Dry Grassland Community
Source: PLoS One. 2016 Jul 8;11(7):e0158925. doi: 10.1371/journal.pone.0158925 (PMC4938501; doi:10.1371/journal.pone.0158925)
Supplement: S2 Table — Significant values (p ≤ 0.05) are in bold. Df Error = 48. For significant effect of soil type for species richness, F and G indicates higher value in the soil from abandoned field and grassland, respectively. For significant effect of fungicide, C and F indicates higher value in the soil from control and fungicide treated plots, respectively. (DOCX) [file pone.0158925.s003.docx]

S3 Table. The effect of soil type, fungicide and their interaction on plant species richness and plant species composition in the experiment. Significant values (p ≤ 0.05) are in bold. Df Error = 48. For significant effect of soil type for species richness, F and G indicates higher value in the soil from abandoned field and grassland, respectively. For significant effect of fungicide, C and F indicates higher value in the soil from control and fungicide treated plots, respectively.

|  |  |  | Covar. | Soil | Fungicid | Year | Soil × fungicid | Soil × year | Fungicide × year | Soil × fungicid × year |
| --- | --- | --- | --- | --- | --- | --- | --- | --- | --- | --- |
| Species richness |  | F |  | 0.15 | **74.64** | **34.42** | **3.99** | **6.00** | **6.00** | 0.20 |
|  |  | p |  | 0.701 | **<0.001 C** | **<0.001** | **0.051** | **0.018** | **0.018** | 0.654 |
|  |  | F | Soil comp. | 0.02 | **66.16** | 0.01 | 0.77 | 1.90 | **6.23** | 0.25 |
|  |  | p |  | 0.900 | **<0.001 C** | 0.915 | 0.386 | 0.176 | **0.017** | 0.621 |
| Species comp. | No stand. | Prop. var. |  | **0.343** | **0.095** | **0.1105** | **0.025** | **0.0543** | 0.0069 | 0.0097 |
|  |  | p |  | **0.002** | **0.002** | **0.002** | **0.02** | **0.002** | 0.444 | 0.184 |
|  |  | Prop. var. | Soil comp. |  | **0.024** |  | 0.003 |  | 0.007 | 0.008 |
|  |  | p |  |  | **0.02** |  | 0.91 |  | 0.47 | 0.354 |
|  | Stand. by sample | Prop. var. |  | **0.082** | **0.2292** | **0.0534** | 0.0275 | **0.0153** | **0.0226** | **0.0176** |
|  |  | p |  | **0.006** | **0.002** | **0.002** | 0.07 | **0.092** | **0.016** | **0.032** |
|  |  | Prop. var. | Soil comp. |  | **0.178** |  | 0.013 |  | 0.022 | 0.014 |
|  |  | p |  |  | **0.002** |  | 0.39 |  | 0.094 | 0.35 |
