# Supplementary material for: Roles of Arbuscular Mycorrhizal Fungi and Soil Abiotic Conditions in the Establishment of a Dry Grassland Community
Source: PLoS One. 2016 Jul 8;11(7):e0158925. doi: 10.1371/journal.pone.0158925 (PMC4938501; doi:10.1371/journal.pone.0158925)
Supplement: S3 Table — (DOCX) [file pone.0158925.s004.docx]

S4 Table. Primary data showing number of species in pots of the different treatments in the two years.

| Pot no. | Year | Grassland soil | Fungicide | No. species |
| --- | --- | --- | --- | --- |
| 1 | 1 | 1 | 0 | 24 |
| 1 | 2 | 1 | 0 | 34 |
| 2 | 1 | 1 | 1 | 24 |
| 2 | 2 | 1 | 1 | 20 |
| 3 | 1 | 0 | 0 | 28 |
| 3 | 2 | 0 | 0 | 26 |
| 4 | 1 | 0 | 1 | 27 |
| 4 | 2 | 0 | 1 | 11 |
| 5 | 1 | 0 | 0 | 28 |
| 5 | 2 | 0 | 0 | 26 |
| 6 | 1 | 0 | 1 | 21 |
| 6 | 2 | 0 | 1 | 9 |
| 7 | 1 | 1 | 0 | 26 |
| 7 | 2 | 1 | 0 | 25 |
| 8 | 1 | 1 | 1 | 26 |
| 8 | 2 | 1 | 1 | 17 |
| 9 | 1 | 1 | 0 | 33 |
| 9 | 2 | 1 | 0 | 30 |
| 10 | 1 | 1 | 1 | 18 |
| 10 | 2 | 1 | 1 | 13 |
| 11 | 1 | 0 | 0 | 25 |
| 11 | 2 | 0 | 0 | 18 |
| 12 | 1 | 0 | 1 | 30 |
| 12 | 2 | 0 | 1 | 13 |
| 13 | 1 | 0 | 0 | 25 |
| 13 | 2 | 0 | 0 | 19 |
| 14 | 1 | 0 | 1 | 18 |
| 14 | 2 | 0 | 1 | 9 |
| 15 | 1 | 1 | 0 | 28 |
| 15 | 2 | 1 | 0 | 31 |
| 16 | 1 | 1 | 1 | 14 |
| 16 | 2 | 1 | 1 | 12 |
| 17 | 1 | 1 | 0 | 31 |
| 17 | 2 | 1 | 0 | 19 |
| 18 | 1 | 1 | 1 | 19 |
| 18 | 2 | 1 | 1 | 8 |
| 19 | 1 | 0 | 0 | 30 |
| 19 | 2 | 0 | 0 | 25 |
| 20 | 1 | 0 | 1 | 27 |
| 20 | 2 | 0 | 1 | 12 |
| 21 | 1 | 0 | 0 | 30 |
| 21 | 2 | 0 | 0 | 21 |
| 22 | 1 | 0 | 1 | 22 |
| 22 | 2 | 0 | 1 | 11 |
| 23 | 1 | 1 | 0 | 29 |
| 23 | 2 | 1 | 0 | 22 |
| 24 | 1 | 1 | 1 | 11 |
| 24 | 2 | 1 | 1 | 7 |
| 25 | 1 | 1 | 0 | 31 |
| 25 | 2 | 1 | 0 | 30 |
| 26 | 1 | 1 | 1 | 23 |
| 26 | 2 | 1 | 1 | 16 |
| 27 | 1 | 0 | 0 | 33 |
| 27 | 2 | 0 | 0 | 22 |
| 28 | 1 | 0 | 1 | 25 |
| 28 | 2 | 0 | 1 | 18 |
