# Supplementary material for: Roles of Arbuscular Mycorrhizal Fungi and Soil Abiotic Conditions in the Establishment of a Dry Grassland Community
Source: PLoS One. 2016 Jul 8;11(7):e0158925. doi: 10.1371/journal.pone.0158925 (PMC4938501; doi:10.1371/journal.pone.0158925)
Supplement: S4 Table — Df Error = 48 for all tests. Significant values (p ≤ 0.05) are in bold. Dev. stands for Deviance. For significant effect of soil type, F and G indicates higher value in the soil from abandoned field and grassland, respectively. For significant effect of fungicide, C and F indicates higher value in the soil from control and fungicide treated plots, respectively. (DOCX) [file pone.0158925.s005.docx]

S5 Table. The effect of soil type, fungicide and year on mycorrhizal inoculation potential of the soil, on chemical composition of the soil and on nutrient content of biomass of *S. verticilata*. Df Error = 48 for all tests. Significant values (p ≤ 0.05) are in bold. Dev. stands for Deviance. For significant effect of soil type, F and G indicates higher value in the soil from abandoned field and grassland, respectively. For significant effect of fungicide, C and F indicates higher value in the soil from control and fungicide treated plots, respectively.

|  |  |  | Soil | Fungicide | Year | Soil × fungicide | Soil × year | Fungicide × year | Soil × fungicide × year |
| --- | --- | --- | --- | --- | --- | --- | --- | --- | --- |
| Soil | MIP | Dev. | **133.68** | **791.18** | <0.001 | 21.44 | <0.001 | <0.001 | <0.001 |
|  |  | p | **0.001 F** | **<0.001 C** | 0.998 | 0.169 | 0.998 | 0.996 | 0.997 |
|  | Total carbon | F | **4523.76** | 0.23 | **69.78** | **5.69** | 2.23 | 0.09 | 0.61 |
|  |  | p | **<0.001 G** | 0.633 | **<0.001** | **0.021** | 0.142 | 0.769 | 0.437 |
|  | pH | F | **262.57** | **45.63** | 1.06 | **22.42** | 2.27 | 1.28 | 1.87 |
|  |  | p | **<0.001 G** | **<0.001 C** | 0.308 | **<0.001** | 0.139 | 0.265 | 0.178 |
|  | Nitrogen | F | **1293.47** | **25.58** | **34.82** | **15.47** | **108.08** | 0.71 | 0.71 |
|  |  | p | **<0.001 F** | **<0.001 F** | **<0.001** | **<0.001** | **<0.001** | 0.403 | 0.403 |
|  | Carbon in carbonates | F | **24947.59** | 0.06 | **111.35** | 0.03 | **4.71** | 0.12 | 0.08 |
|  |  | p | **<0.001 G** | 0.811 | **<0.001** | 0.868 | **0.035** | 0.726 | 0.782 |
|  | Organic carbon | F | **20.87** | 0.33 | **13.31** | **5.83** | **5.91** | 0.2 | 0.44 |
|  |  | p | **<0.001 F** | 0.569 | **0.001** | **0.02** | **0.019** | 0.654 | 0.512 |
|  | Phosphorus | F | **19.57** | 0.19 | 13.78 | 0.03 | 0.97 | 0.03 | 2.13 |
|  |  | p | **<0.001 F** | 0.666 | **0.001** | 0.867 | 0.331 | 0.862 | 0.151 |
|  | C/N | F | **781.78** | **5.11** | **77.28** | **4.78** | **92.36** | 0.95 | 1.17 |
|  |  | p | **<0.001 G** | **0.028 C** | **0** | **0.034** | **<0.001** | 0.334 | 0.284 |
| Biomass of *Salvia verticilata* | Phosphorus | F | 0.55 | **22.01** | **9.1** | 2.42 | 0.58 | 1.25 | 0.41 |
|  |  | p | 0.462 | **<0.001 C** | **0.004** | 0.126 | 0.448 | 0.27 | 0.528 |
|  | Nitrogen | F | 0.37 | **63.06** | **7.3** | 3.48 | 0.07 | **5.73** | 1.82 |
|  |  | p | 0.545 | **<0.001 F** | **0.01** | 0.068 | 0.791 | **0.021** | 0.184 |
|  | C/N | F | 0.01 | **50.44** | 3.09 | 2.67 | 0 | 0.85 | 0.68 |
|  |  | p | 0.912 | **<0.001 C** | 0.085 | 0.109 | 0.952 | 0.362 | 0.414 |
