# Supplementary material for: Roles of Arbuscular Mycorrhizal Fungi and Soil Abiotic Conditions in the Establishment of a Dry Grassland Community
Source: PLoS One. 2016 Jul 8;11(7):e0158925. doi: 10.1371/journal.pone.0158925 (PMC4938501; doi:10.1371/journal.pone.0158925)
Supplement: S5 Table — (DOCX) [file pone.0158925.s006.docx]

S6 Table. Primary data showing root colonization in the mycorrhizal inoculation assessment in the pots of the different treatments.

| Pot no. | Year | Grassland soil | Fungicide | Prop. colonized roots |
| --- | --- | --- | --- | --- |
| 1 | 2009 | 1 | 1 | 0.0176722 |
| 2 | 2009 | 1 | 1 | 0.0385164 |
| 3 | 2009 | 1 | 1 | 0.005992 |
| 4 | 2009 | 1 | 1 | 0.0133028 |
| 5 | 2009 | 1 | 1 | 0.0289487 |
| 6 | 2009 | 1 | 1 | 0.0251116 |
| 7 | 2009 | 1 | 1 | 0.0104287 |
| 8 | 2009 | 1 | 0 | 0.0538793 |
| 9 | 2009 | 1 | 0 | 0.0153739 |
| 10 | 2009 | 1 | 0 | 0.0385588 |
| 11 | 2009 | 1 | 0 | 0.0785311 |
| 12 | 2009 | 1 | 0 | 0.0695995 |
| 13 | 2009 | 1 | 0 | 0.0933063 |
| 14 | 2009 | 1 | 0 | 0.0637329 |
| 15 | 2009 | 0 | 1 | 0.0301669 |
| 16 | 2009 | 0 | 1 | 0.0220994 |
| 17 | 2009 | 0 | 1 | 0.0248918 |
| 18 | 2009 | 0 | 1 | 0.0313589 |
| 19 | 2009 | 0 | 1 | 0.0553042 |
| 20 | 2009 | 0 | 1 | 0.0366667 |
| 21 | 2009 | 0 | 1 | 0.0510753 |
| 22 | 2009 | 0 | 0 | 0.0513069 |
| 23 | 2009 | 0 | 0 | 0.0973828 |
| 24 | 2009 | 0 | 0 | 0.0631229 |
| 25 | 2009 | 0 | 0 | 0.0761421 |
| 26 | 2009 | 0 | 0 | 0.0932999 |
| 27 | 2009 | 0 | 0 | 0.1170047 |
| 28 | 2009 | 0 | 0 | 0.0435424 |
| 1 | 2010 | 1 | 1 | 0.0176722 |
| 2 | 2010 | 1 | 1 | 0.0385164 |
| 3 | 2010 | 1 | 1 | 0.005992 |
| 4 | 2010 | 1 | 1 | 0.0133028 |
| 5 | 2010 | 1 | 1 | 0.0289487 |
| 6 | 2010 | 1 | 1 | 0.0251116 |
| 7 | 2010 | 1 | 1 | 0.0104287 |
| 8 | 2010 | 1 | 0 | 0.0538793 |
| 9 | 2010 | 1 | 0 | 0.0153739 |
| 10 | 2010 | 1 | 0 | 0.0385588 |
| 11 | 2010 | 1 | 0 | 0.0785311 |
| 12 | 2010 | 1 | 0 | 0.0695995 |
| 13 | 2010 | 1 | 0 | 0.0933063 |
| 14 | 2010 | 1 | 0 | 0.0637329 |
| 15 | 2010 | 0 | 1 | 0.0301669 |
| 16 | 2010 | 0 | 1 | 0.0220994 |
| 17 | 2010 | 0 | 1 | 0.0248918 |
| 18 | 2010 | 0 | 1 | 0.0313589 |
| 19 | 2010 | 0 | 1 | 0.0553042 |
| 20 | 2010 | 0 | 1 | 0.0366667 |
| 21 | 2010 | 0 | 1 | 0.0517711 |
| 22 | 2010 | 0 | 0 | 0.0513069 |
| 23 | 2010 | 0 | 0 | 0.0973828 |
| 24 | 2010 | 0 | 0 | 0.0631229 |
| 25 | 2010 | 0 | 0 | 0.0761421 |
| 26 | 2010 | 0 | 0 | 0.0932999 |
| 27 | 2010 | 0 | 0 | 0.1170047 |
| 28 | 2010 | 0 | 0 | 0.0435424 |
