# Supplementary material for: Roles of Arbuscular Mycorrhizal Fungi and Soil Abiotic Conditions in the Establishment of a Dry Grassland Community
Source: PLoS One. 2016 Jul 8;11(7):e0158925. doi: 10.1371/journal.pone.0158925 (PMC4938501; doi:10.1371/journal.pone.0158925)
Supplement: S6 Table — Significant values (p ≤ 0.05) are in bold. For significant effect of soil type, F and G indicates higher value in the soil from abandoned field and grassland, respectively. For significant effect of fungicide, C and F indicates higher value in the soil from control and fungicide treated plots, respectively. (DOCX) [file pone.0158925.s007.docx]

S7 Table. The effect of soil type, fungicide and their interaction on root colonization of plants in the experiment. Significant values (p ≤ 0.05) are in bold. For significant effect of soil type, F and G indicates higher value in the soil from abandoned field and grassland, respectively. For significant effect of fungicide, C and F indicates higher value in the soil from control and fungicide treated plots, respectively.

|  |  | Soil | Fungicide | Soil x fungicide | df Error |
| --- | --- | --- | --- | --- | --- |
| *B. pinnatum* | Deviance | **39.4** | 0.77 | 2.99 | 53 |
|  | p | **<0.001 F** | 0.625 | 0.335 |  |
| *B. erectus* | Deviance | 0.25 | **228.06** | 17.2 | 34 |
|  | p | 0.857 | **<0.001 C** | 0.137 |  |
| *B. media* | Deviance | **33.94** | **51.45** | **47.17** | 66 |
|  | p | **0.051 G** | **0.016 C** | **0.022** |  |
| *C. jacea* | Deviance | 3.93 | **1076.67** | 7.69 | 76 |
|  | p | 0.634 | **<0.001 C** | 0.505 |  |
| *C. scabiosa* | Deviance | 38.24 | **126.55** | 40.85 | 29 |
|  | p | 0.08 | **0.001 C** | 0.071 |  |
| *F. rupicola* | Deviance | 59.25 | **100.18** | 22.19 | 38 |
|  | p | 0.146 | **0.059 C** | 0.374 |  |
| *S. nemorosa* | Deviance | **93.16** | **182.43** | 0.12 | 32 |
|  | p | **0.047 G** | **0.005 C** | 0.944 |  |
| *S. verticilata* | Deviance | 2.4 | **578.48** | 0.77 | 85 |
|  | p | 0.681 | **<0.001 C** | 0.816 |  |
| *S. hispanica* | Deviance | **68.98** | **158.14** | 14.91 | 41 |
|  | p | **0.021 F** | **<0.001 C** | 0.283 |  |
