# Supplementary material for: Roles of Arbuscular Mycorrhizal Fungi and Soil Abiotic Conditions in the Establishment of a Dry Grassland Community
Source: PLoS One. 2016 Jul 8;11(7):e0158925. doi: 10.1371/journal.pone.0158925 (PMC4938501; doi:10.1371/journal.pone.0158925)
Supplement: S7 Table — The values are mean±SE of proportion of roots colonized. (DOCX) [file pone.0158925.s008.docx]

S8 Table. Proportional root colonization of plants in the experiment. The values are mean±SE of proportion of roots colonized.

|  | Grassland | | Field | |
| --- | --- | --- | --- | --- |
| Species | Fungicide | Control | Fungicide | Control |
| *B. pinnatum* | 0.608±0.020 | 0.588±0.023 | 0.661±0.014 | 0.676±0.013 |
| *B. media* | 0.540±0.032 | 0.582±0.013 | 0.444±0.039 | 0.589±0.029 |
| *B. erectus* | 0.526±0.058 | 0.649±0.025 | 0.460±0.026 | 0.697±0.025 |
| *C. jacea* | 0.598±0.039 | 0.827±0.022 | 0.524±0.058 | 0.839±0.018 |
| *C. scabiosa* | 0.584±0.059 | 0.597±0.092 | 0.493±0.081 | 0.761±0.026 |
| *F. rupicola* | 0.412±0.080 | 0.491±0.046 | 0.262±0.106 | 0.441±0.060 |
| *S. nemorosa* | 0.305±0.072 | 0.566±0.076 | 0.429±0.088 | 0.668±0.047 |
| *S. verticilata* | 0.507±0.050 | 0.712±0.025 | 0.542±0.023 | 0.711±0.022 |
| *S. hispanica* | 0.390±0.068 | 0.675±0.074 | 0.567±0.059 | 0.712±0.046 |
