# Supplementary material for: Roles of Arbuscular Mycorrhizal Fungi and Soil Abiotic Conditions in the Establishment of a Dry Grassland Community
Source: PLoS One. 2016 Jul 8;11(7):e0158925. doi: 10.1371/journal.pone.0158925 (PMC4938501; doi:10.1371/journal.pone.0158925)
Supplement: S8 Table — The values are mean±SE. (DOCX) [file pone.0158925.s009.docx]

S9 Table. Content of phosphorus, nitrogen and carbon to nitrogen ratio in biomass of selected species grown in the soil from the abandoned field and grassland, with and without fungicide application. The values are mean±SE.

|  |  |  | Field | | Grassland | |
| --- | --- | --- | --- | --- | --- | --- |
| Biomass content | Species | Year | Control | Fungicide | Control | Fungicide |
| P | *B. pinnatum* | 2009 | 2841±117 | 1234±112 | 1888±186 | 906.±90.1 |
|  | *C. jacea* | 2010 | 3732±173 | 4225±419 | 3085±111 | 2748±469 |
|  | *S. verticilata* | 2009 | 3186±497 | 2171±120 | 4225±783 | 1978±333 |
|  |  | 2010 | 4114±264 | 3368±171 | 4365±374 | 3103±188 |
| N | *B. pinnatum* | 2009 | 2.33±0.0 | 3.32±0.1 | 2.63±0.1 | 3.03±0.2 |
|  | *C. jacea* | 2010 | 2.45±0.1 | 4.61±0.2 | 2.23±0.1 | 4.30±0.1 |
|  | *S. verticilata* | 2009 | 2.86±0.2 | 3.24±0.1 | 2.48±0.2 | 3.91±0.3 |
|  |  | 2010 | 2.73±0.1 | 4.35±0.1 | 2.70±0.2 | 4.49±0.1 |
| C/N | *B. pinnatum* | 2009 | 18.7±0.5 | 13.1±0.6 | 16.5±1.2 | 14.3±1.3 |
|  | *C. jacea* | 2010 | 17.7±1.0 | 9.58±0.6 | 19.1±0.9 | 10.0±0.3 |
|  | *S. verticilata* | 2009 | 15.6±0.9 | 12.6±0.7 | 17.6±1.4 | 10.7±1.3 |
|  |  | 2010 | 15.5±0.9 | 9.77±0.3 | 16.3±1.9 | 9.26±0.2 |
