# Supplementary material for: Roles of Arbuscular Mycorrhizal Fungi and Soil Abiotic Conditions in the Establishment of a Dry Grassland Community
Source: PLoS One. 2016 Jul 8;11(7):e0158925. doi: 10.1371/journal.pone.0158925 (PMC4938501; doi:10.1371/journal.pone.0158925)
Supplement: S9 Table — The values are mean±SE. (DOCX) [file pone.0158925.s010.docx]

S10 Table. Content of nitrogen, total and organic carbon and carbon in carbonates, phosphorus, pH and C/N ratio in soil from the abandoned field and grassland with and without fungicide application. The values are mean±SE.

|  |  | pH | N | C-total | C-carbon. | C-organic | P | C/N |
| --- | --- | --- | --- | --- | --- | --- | --- | --- |
| Field | Control | 7.77±0.04 | 0.09±0.01 | 0.78±0.08 | 0.11±0.02 | 0.66±0.06 | 6.10±0.487 | 8.772±1.15 |
|  | Fungicide | 7.35±0.04 | 0.12±0.01 | 0.90±0.06 | 0.11±0.03 | 0.78±0.05 | 5.88±0.474 | 8.122±0.93 |
| Grassland | Control | 8.18±0.03 | 0.01±0.00 | 3.73±0.05 | 3.15±0.04 | 0.57±0.03 | 4.38±0.351 | 271.0±25.1 |
|  | Fungicide | 8.11±0.03 | 0.01±0.00 | 3.65±0.07 | 3.15±0.04 | 0.50±0.04 | 4.28±0.337 | 232.3±25.3 |
